# Functional neuroimaging and withdrawal of life-sustaining treatment from vegetative patients

**DOI:** 10.1136/jme.2008.029165

**Published:** 2009-07-23

**Authors:** D J Wilkinson, G Kahane, M Horne, J Savulescu

**Affiliations:** 1 Oxford Uehiro Centre for Practical Ethics, University of Oxford, Oxford, UK; 2 The Ethox Centre, University of Oxford, Oxford, UK; 3 The Oxford Centre for Neuroethics, University of Oxford, Oxford, UK; 4 Neural Injury and Repair Group, Howard Florey Institute and the Centre for Neuroscience, The University of Melbourne, Melbourne, Australia

## Abstract

Recent studies using functional magnetic resonance imaging of patients in a vegetative state have raised the possibility that such patients retain some degree of consciousness. In this paper, the ethical implications of such findings are outlined, in particular in relation to decisions about withdrawing life-sustaining treatment. It is sometimes assumed that if there is evidence of consciousness, treatment should not be withdrawn. But, paradoxically, the discovery of consciousness in very severely brain-damaged patients may provide more reason to let them die. Although functional neuroimaging is likely to play an increasing role in the assessment of patients in a vegetative state, caution is needed in the interpretation of neuroimaging findings.

Following severe brain injury, some patients emerge from coma to a vegetative state.^1^ They appear awake but unaware of themselves or of their environment. The likelihood of recovery is extremely small when the vegetative state persists for more than 12 months following traumatic brain injury, or 3 months with a non-traumatic cause.^2^ Beyond this point the vegetative state is often referred to as “permanent”. In such patients life-sustaining treatment, including artificial nutrition and hydration, is sometimes withdrawn. Legal decisions relating to treatment withdrawal have been highly controversial in cases such as those of Karen Quinlan, Tony Bland or Terri Schiavo.^3^ ^4^ Nevertheless, when the diagnosis of a permanent vegetative state is clear, many believe that it is morally permissible to let such patients die.^5^

Concern is sometimes expressed about allowing patients in a vegetative state to die, because of the possibility that they might actually be conscious despite the absence of evidence of awareness. The diagnosis of vegetative state is based upon the observation of behavioural responses to stimuli, and assessment of such patients can be extremely difficult.^6^ Recently published papers using functional magnetic resonance imaging (fMRI) may shed some light on this question. There is evidence on fMRI of islands of preserved cognitive function,^7^ including processing of spoken words^8^ in some patients in a vegetative state. There were specific changes on fMRI seen in two patients in a vegetative state asked to imagine themselves performing an activity such as playing tennis or walking through the rooms of a house, similar to those observed in normal conscious individuals.^9^ ^10^ Those two patients, who had been in a vegetative state for several months after traumatic brain injuries, subsequently improved clinically to the point at which they inconsistently responded to stimuli^9^ and met the criteria for a less severe disorder of consciousness (a minimally conscious state; MCS).^11^ It is often assumed that the presence of consciousness makes the withdrawal of life-sustaining treatment problematical or even unjustifiable.^12^ The fMRI findings have thus been claimed to have profound implications for treatment decisions.^9^

It was suggested at the time that both Terri Schiavo,^13^ and a more recent patient in the UK,^4^ undergo fMRI before decisions about withdrawal of artificial nutrition. Although in both cases the courts rejected this suggestion, the question of the role of neuroimaging in assessment and decision-making for patients in an apparent vegetative state is likely to be raised again.^14^ Consciousness is a difficult and contentious concept. Elsewhere we have elaborated on the potential moral significance of different forms of consciousness in severely brain-damaged patients.^15^ For this paper we have assumed consciousness to involve at least minimal sentience and the capacity to suffer. Here we wish to address the ethical question raised by these advances in neuroimaging: if we have evidence of consciousness in a patient previously thought to be in a vegetative state, is it permissible to withdraw life-sustaining treatment? We review the ethical basis for withdrawal of life-sustaining treatment, and the possible relevance of neuroimaging findings for such decisions. Importantly, evidence of consciousness from neuroimaging may provide reasons both for and against continuing to keep such patients alive.

## ARE THEY CONSCIOUS? POSSIBLE INTERPRETATIONS OF NEUROIMAGING

Before considering how neuroimaging may affect treatment withdrawal decisions, it is worthwhile outlining different possible interpretations of the neuroimaging findings. The researchers who reported fMRI changes in response to instructions were confident that their results established that the patient was genuinely conscious of herself and her surroundings.^10^ This conclusion may be premature.^16^ The responses to instructions might have been automatic rather than evidence of conscious volition.^17^ ^18^ For example, unconscious patients under anaesthesia have evidence of cognitive processing, including complex processing of language.^19^ It therefore remains unclear whether reported fMRI responses do in fact represent evidence of consciousness. Further research is underway, and may resolve this empirical matter. However it seems that there are three main possibilities (box 1).

**Box 1 Possible interpretations of neuroimaging evidence of “consciousness”**

- No consciousness: patients who demonstrate evidence of language processing, or patterns of brain activation in response to instruction, have no genuine awareness of these stimuli; these are merely automatic responses.
- Minimal consciousness: although lacking sustained, reproducible, voluntary or purposeful behavioural responses to stimuli, some patients in a vegetative state respond to stimuli with functional changes that are detectable with neuroimaging. They may have intermittent, incomplete consciousness of themselves and their surroundings. These patients may represent an overlap category with MCS or they may be emerging from a vegetative state to MCS.
- Full consciousness: patients who are able to obey instructions and demonstrate patterns of activation on functional imaging may be completely aware of their surroundings. Some patients who appear to be in a vegetative state may actually be in a complete form of the locked-in syndrome (LIS).

It should be noted that negative findings with functional neuroimaging are also open to interpretation. Failure to observe changes in response to instructions or stimuli might be due to the patient failing to hear, remember or understand instructions, or to being asleep.^20^ Just as repeated behavioural testing is required to make a diagnosis of a vegetative state, repeated testing with functional neuroimaging may be necessary to reduce the risk of false negative test results.^9^ But how should neuroimaging findings affect decisions about life-sustaining treatment?

## THE ETHICAL BASIS FOR WITHDRAWAL OF LIFE-SUSTAINING TREATMENT IN A VEGETATIVE STATE

Decisions to continue or withdraw life-sustaining treatment are influenced by a number of factors, reflecting the ethical principles that are often drawn upon to guide medical care.^21^^–^24 First, the patient’s wishes may be known, for example in the form of an advance directive, and in accordance with the principle of respect for autonomy this may guide decisions either to continue or to discontinue treatment. Second, ongoing treatment may be judged to be futile on the basis of the very low likelihood of significant recovery, and may risk harming the patient. Such concerns can be based upon the principle of non-maleficence. Third, consideration may be given to the best interests of the patient, and whether the benefits outweigh the burdens of treatment (the principle of beneficence). As patients in a vegetative state have generally been thought to lack all awareness, some have argued that they do not have interests as such, and hence providing life-sustaining treatment cannot be in their interests.^5^ ^25^ ^26^ Finally, considerations of distributive justice may warrant withdrawal of treatment when such treatment (for example intensive care) has finite availability and other individuals have greater prospects of recovery.^21^

### Arguments in favour of continuing life-sustaining treatment

The detection of possible consciousness using fMRI or other forms of imaging might have relevance to considerations of the patient’s wishes and autonomy. This would depend upon the wording of advance directives, or upon how patients had expressed to family and friends their wishes for future treatment.^14^ Given that few patients are likely to have explicitly considered the possibility of being in a vegetative state, MCS or LIS, it may be hard to know what the patient would have wanted.^14^ One intriguing possibility would be to use fMRI to communicate with patients who have no other means of expression.^9^ ^20^ ^27^ It may give such patients the chance to voice their own opinion about the continuation or withdrawal of life-sustaining treatment. At present this possibility remains speculative.

Second, fMRI changes may imply a better prognosis for patients in a vegetative state and lead to reconsideration of the futility of ongoing treatment. A recent review of published case reports and small case series suggested that patients in a vegetative state studied in the first months who showed activation of higher level associative cortices on neuroimaging were more likely subsequently to improve clinically.^28^ It is even possible that neuroimaging could detect the rare patient in a “permanent” vegetative state who is able to make a significant recovery. Alternatively, by identifying patients with islands of preserved cognitive function, neuroimaging may direct the provision of treatment such as deep-brain stimulation^29^ leading to greater recovery.^9^

Finally, consciousness may be relevant to the interests of the patient. If the patient is minimally conscious or fully conscious, they have interests that may be furthered by providing life-sustaining treatment. They may be aware of their surroundings, of family and friends, and may be able to take pleasure in those things. There is no ethical or legal consensus about the withdrawal of life-sustaining treatment from children or adults in MCS or LIS.^23^ ^30^ If patients can be shown to be conscious or even self-conscious, this may confer moral status,^15^ ^31^ which would make a difference to an appraisal of whether life-sustaining treatment should continue.

### Arguments in favour of discontinuing life-sustaining treatment

On the other hand, neuroimaging findings may not predict meaningful recovery. The reported findings^8^ ^10^ ^28^ are preliminary, and the degree of recovery often relatively minor (from vegetative state to MCS). This recovery has occurred soon after traumatic brain injury (ie, within the time period when recovery is not unusual). There are no reports of patients in a vegetative state identified as “conscious” by functional neuroimaging making a recovery to functional independence, and no patients meeting the criteria for a permanent vegetative state have shown these changes on fMRI.

Second, minimal degrees of consciousness may not be a benefit to the patient. People with MCS following brain injury are severely impaired, with only some evidence of voluntary movement (for example patients may track an object with their eyes), but without the capacity for functional communication.^32^ Patients in a MCS seem to have only fleeting, fragmented awareness of their environment. Although there is a greater chance of recovery than with a vegetative state, most patients in MCS for more than 12 months remain severely disabled.^11^ Minimally conscious patients remain bed-bound, doubly incontinent, dependent and limited in their ability to interact.^1^ It is not possible to know what being in a MCS would be like;^27^ however, in one important way it may be considerably worse than being in a vegetative state.^23^ ^30^ A recent positron emission tomography study of minimally conscious patients strongly suggests that they experience pain and suffer,^33^ although they are largely unable to tell us.^12^ If such patients suffer they can be harmed by continuing treatment; there may be stronger reasons in terms of non-maleficence and the best interests of the patient to allow them to die. This must be balanced against the possibility of having positive experiences, and the greater uncertainty about prognosis for such patients compared with those in a permanent vegetative state.^27^

Third, consciousness does not preclude withdrawal of life-sustaining treatment. It is ethical to withdraw life-sustaining treatment even from patients who are fully conscious, for example when the patient requests it, when the treatment is futile, or when the burdens of treatment outweigh their benefits.^24^ Neuroimaging might indicate that a patient who appears to be in a vegetative state is actually fully conscious and “locked-in”. Whereas it might be thought that we are morally required to do our best to preserve the life of a patient in this state, some might ask whether such a life is worse than death. A number of patients with LIS express a desire not to go on living^3^ and some request euthanasia.^34^ On the other hand the capacity of humans to adapt to their condition, no matter how adverse, has been amply documented in other contexts. Indeed, the self-scored perception of mental wellbeing in one survey of patients with LIS was not significantly lower than that of age-matched normal subjects.^35^ However, surveys of patients able to communicate may not be relevant to a more severely affected population of patients with full LIS and an apparent vegetative state. Many may also have been in such a state for a long time. Their situation might be compared unfavourably with the worst form of solitary confinement in prison. It is not necessarily clear, on the basis of the best interests of such patients, that life-sustaining treatment should be continued. This is a subject that requires further active debate.

Finally, considerations of medical utility^21^ and distributive justice may warrant the prioritisation of scarce medical resources to other patients with greater prospect of benefit even if consciousness is detected with neuroimaging, especially in cases in which the life sustained contains significant suffering.

## CONCLUSIONS

It is early days for fMRI in patients in a vegetative state and reported findings need to be interpreted with caution.^36^ There are well-defined methodological limitations of fMRI.^37^ Techniques for interrogation of consciousness such as those discussed in this paper are available only in a small number of centres in the context of research studies. It is not yet clear whether fMRI changes represent automatic responses or whether they represent minimal or even full consciousness. There is very limited evidence for the prognostic relevance of fMRI, or for its use in identifying patients amenable to therapy.

fMRI has already been called for in legal disputes about treatment withdrawal from patients in a vegetative state, and is likely to be called upon for this purpose increasingly.^14^ Within existing legal frameworks it may have some relevance for such decisions.^38^ Yet it will be important to avoid jumping to conclusions about whether such patients are conscious or whether treatment should be continued on the basis of neuroimaging. Whether such patients are conscious, minimally or fully, may be relevant to their interests, although this may provide reasons both for and against continuing life-sustaining treatment.

Advances in neuroimaging may inform management, but do not and will not in themselves settle ethical questions around the treatment of people with severe neurological disorders. Such decisions require both good science and good ethical judgement. Paradoxically, the discovery of consciousness in very severely brain-damaged patients may give us more reason to discontinue life-sustaining treatment than to continue it.
